# Risk of Severe SARS-CoV-2 Infection in Patients with Autoimmune Rheumatic Diseases in Qatar: A Cohort Matched Study

**DOI:** 10.5339/qmj.2022.24

**Published:** 2022-06-20

**Authors:** Omar Alsaed, Samar Alemadi, Eman Satti, Karima Becetti, Rawan Saleh, Hadil Ashour, Miral Hamed, Fiaz Alam, Yousef Alrimawi, Joanne Nader, Masautso Chaponda, Basem Awadh, Mohammad Hammoudeh

**Affiliations:** ^1^ Division of Rheumatology, Department of Medicine, Hamad Medical Corporation, Doha, Qatar E-mail: Oalsaed@hamad.qa; ^2^ Division of Infectious Disease, Department of Medicine, Hamad Medical Corporation, Doha, Qatar

**Keywords:** Autoimmune rheumatic disease, Disease-modifying antirheumatic drugs, Poor prognostic factors, Severe acute respiratory syndrome coronavirus 2

## Abstract

Background: It remains unclear whether patients with autoimmune rheumatic diseases (ARDs) are at a higher risk of poor outcomes from a SARS-CoV-2 infection. We evaluated whether patients with an ARDs infected with SARS-CoV-2 were at a higher risk of a poorer outcome than those without an ARDs.

Methods: Patients with an ARDs infected with SARS-CoV-2 were matched to control patients without a known ARDs. Matching was performed according to age ( ± 6 years) and sex at a case-to-control ratio of 1:3. Demographic and clinical data were extracted from the databases and were compared between the two groups. Severe SARS-CoV-2 infection was the primary outcome and was defined as the requirement for oxygen therapy support, the need for invasive or noninvasive mechanical ventilation, or the use of glucocorticoids.

Results: A total of 141 patients with an ARDs were matched to 398 patients who formed the control group. The mean ages (SD) of the ARDs and non-ARDs groups were 44.4 years (11.4) and 43.4 years (12.2). Women accounted for 58.8% of the ARDs group and 56.3% of the control group (p = 0.59). Demographics and comorbidities were balanced between the groups. ARDs included connective tissue disease in 43 (30.3%) patients, inflammatory arthritis in 92 (65.2%), and other ARDs in 8 (5.7%). ARDs medications included biological/targeted synthetic disease-modifying antirheumatic drugs (b/ts-DMARDs) in 28 (15.6%) patients, conventional synthetic DMARDs in 95 (67.4%), and immunosuppressive antimetabolites in 13 (9.2%). The ARDs group had more respiratory and gastrointestinal symptoms related to SARS-CoV-2 infection than the control group (24.8% and 20.6% vs. 10% and 5.3%, respectively; p <  0.001 for both). Severe SARS-CoV-2 infection was more common in the ARDs group than in the control group (14.9% vs. 5.8%; p <  0.001).

Conclusions: In this single-center matched cohort study, patients with an ARDs experienced more respiratory and gastrointestinal symptoms related to SARS-CoV-2 infection and had more severe infection than those from the control group. Therefore, patients with an ARDs require close observation during the coronavirus disease 2019 pandemic.

## Introduction:

Since the World Health Organization (WHO) declared that the severe acute respiratory syndrome coronavirus 2 (SARS-CoV-2) infection is a pandemic, many studies have examined SARS-CoV-2 infection and rheumatic diseases from many perspectives. However, many clinicians remain uncertain whether patients with autoimmune rheumatic diseases (ARDs) are at higher risk of a severe SARS-CoV-2 infection that might require admission to the intensive care unit (ICU) or have higher mortality rates than those without an ARDs. Patients with an ARDs are at higher risk of infection in general, which is usually more severe relative to those in the general population. This is due to immune system dysfunction and the use of immunomodulating and immunosuppressing medications^1-3^. Many factors that affect the outcomes of a SARS-CoV-2 infection have been identified during the pandemic, such as age of >65 years, male sex, diabetes mellitus, hypertension, cardiovascular disease, chronic kidney disease, chronic lung disease, pregnancy, and morbid obesity^4-9^. In addition, the methods used for SARS-CoV-2 screening and tracking in each nation, as well as the quality and capacity of the healthcare system in each country, the SARS-CoV-2 infection treatment regimens, and accuracy of data collection and networking between hospitals in each country play a role in the limited validity and reliability of the information reported regarding SARS-CoV-2 outcomes.

It has been estimated that although the prevalence of SARS-CoV-2 infection in Qatar is among the highest globally, the mortality rate is among the lowest (8,188 per 100,000 and 20.96 per 100,000, respectively)^10^. Older age (adjusted odds ratio [aOR]: 1.041), male sex (aOR: 4.375), diabetes (aOR: 1.698), chronic kidney disease (aOR: 3.590), and a higher body mass index (BMI) (aOR: 1.067) are independently associated with an increased risk of ICU admission^8^. A stratified analysis of ARDs and the use of immunomodulators and immunosuppressants was not possible in this study. In this retrospective matched cohort study, we evaluated whether the presence of an ARDs and the use of rheumatic disease medications were associated with severe SARS-CoV-2 infection compared to the absence of an ARDs. Rather than compiling data from different nations across different health systems, we took advantage of the advanced and integrated tracking and reporting system of SARS-CoV-2 infection in Qatar, and we believe that our data provide an accurate estimate of the true risk associated with SARS-CoV-2 infection in patients with an ARDs. The cumulative data from our region and other studies will help to obtain generalizable information to aid evidence-based decision-making for managing patients with an ARDs throughout the coronavirus disease 2019 (COVID-19) pandemic.

## Methods

### Study aims and design

This single-center retrospective matched cohort study was conducted to evaluate whether patients with an ARDs and infected with SARS-CoV-2 are at a higher risk of poorer outcomes than those without an ARDs.

### Study sample

The medical center where this study was conducted is a contributing center for the COVID-19 Global Rheumatology Alliance Registry. Patients with an ARDs and infected with SARS-CoV-2 and who were treated at the center were identified from this registry^11,12^. These patients were matched with patients without an ARDs who was identified in the center's databases. Data from March 2020 to March 2021 were used in this study. SARS-CoV-2 infection was confirmed using a positive nasopharyngeal polymerase chain reaction (PCR) swab test. Matching was performed according to age ( ± 6 years) and sex at a case-to-control ratio of 1:3.

### Study variables

Patient demographic data and comorbidities, such as diabetes mellitus, hypertension, chronic kidney disease, chronic heart disease, chronic lung disease, smoking status, obesity (BMI ≥  30 kg/m^2^), relative COVID-19 symptoms, medications used for COVID-19 infection, the need for oxygen therapy and invasive or noninvasive ventilation support, and COVID-19 complications were evaluated in the study groups. Additional data were collected for the ARDs group, including medications used for underlying ARDs and level of disease control before the SARS-CoV-2 infection.

### Outcomes

The primary outcome of our study was severe SARS-CoV-2 infection. We defined severe infection as SARS-CoV-2-infected patients receiving either oxygen therapy support, invasive or noninvasive ventilation, or glucocorticoids.

### ARDs and rheumatic disease medications stratification

The ARDs were classified into inflammatory arthritis [rheumatoid arthritis (RA) and spondyloarthropathy (SPA)], connective tissue diseases (CTD) [systemic lupus erythematosus (SLE), Sjogren's disease, inflammatory myositis, systemic sclerosis, mixed connective tissue disease, undifferentiated connective tissue disease, and antiphospholipid syndrome], and others (sarcoidosis, IgG4-related disease, crystal-induced arthropathy, and familial Mediterranean fever). Rheumatic disease medications were classified into conventional synthetic disease-modifying antirheumatic drugs (Cs-DMARDs) (hydroxychloroquine, sulfasalazine, leflunomide, and methotrexate); biological/targeted synthetic DMARDs (b/ts-DMARDS) [tumor necrosis factor inhibitors (TNF-i), interleukin 6 inhibitors, interleukin 12/23 inhibitors, interleukin 17 inhibitors, Janus kinase inhibitor (JAK-i), and rituximab]; and immunosuppressive drugs (azathioprine, mycophenolate, cyclosporine, cyclophosphamide, tacrolimus, and glucocorticoids). The medication regimens were further classified into monotherapy, dual therapy, and triple therapy.

### Statistical analysis

Patients with an ARDs were matched according to age ( ± 6 years) and sex, with three positive SARS-CoV-2 controls taken from the Qatar COVID-19 national database using the “rangejoin” command in Stata Statistical Software Release 16.1 (StataCorp Llc., College Station, TX, USA).

Dichotomous variables are presented as numbers and percentages, and continuous variables are presented as medians and means (SD) The chi-square test was performed to compare the frequency of events between the cases and controls. Binary logistic regression analysis was performed to study the association between the binary dependent variable (severe infection) and the set of independent variables using a logit model. A p-value of < 0.05 was considered significant.

### Ethics approval and consent to participate

The Ethics Committee of Hamad Medical Corp. approved this study (protocol number 01-20-604). The need for informed consent was waived, as data were extracted retrospectively from the medical records and were rendered innominate.

## Results

### Demographic and baseline clinical characteristics

During the study period, 141 patients with an ARDs and a confirmed SARS-CoV-2 infection were matched with 398 SARS-CoV-2-infected controls without an ARDs. Both study groups were balanced in terms of baseline characteristics, including age, sex, and comorbidities. Females were slightly more predominant in both study groups (58.8% and 56.3% in the case and control groups, respectively). The mean ages (SD) were 44.4 years (11.4) and 43.4 years (12.2) in the case and control groups, respectively. Table 1 summarizes the baseline demographic and clinical characteristics of the study groups.

### ARDs baseline characteristics

A wide variation in ARDs was observed in our cohort. The inflammatory arthritis subgroup comprised 65.2% of the cases. RA was the most common disease in the inflammatory arthritis subgroup comprised of 57 patients (40.4%), followed by spondyloarthropathy (SPA) with 26 patients (psoriatic arthritis, 9.7%; axial SPA, 7.7%; and reactive arthritis, 1.4%). The CTD subgroup constituted 30.3% of the cases. SLE was the most common condition in the CTD subgroup (16 patients [11.3%]), followed by Sjogren's syndrome (8 patients [5.6%]). Fifteen patients (10.6%) had antiphospholipid syndrome. The frequencies of other CTDs and other ARDs are shown in Table 2. At the time of the SARS-CoV-2 infection, ARDs was either in remission or had low disease activity in the majority of patients (85.1%), whereas 12.8% had moderate-to-high disease activity. Disease activity status was unknown in 2% of patients.

### Immunomodulators and immunosuppressors in the ARDs group

As shown in Table 2, 55% of the patients in the ARDs group were on at least one immunosuppressive or immunomodulating agent; 21% were on dual therapy, and 7% were on triple therapy ( ≥ 3 drugs) at the time of the SARS-CoV-2 infection. Hydroxychloroquine and methotrexate were the most commonly used Cs-DMARDs in 48 (34%) and 45 (31.9%) patients, respectively. Furthermore, 28 (15.6%) patients were on b/ts-DMARDs: 20 on TNF-i, 5 on rituximab, and 3 on JAK-i.

### SARS-CoV-2 infection characteristics of the study groups and outcomes

Table 3 compares the clinical features of the SARS-CoV-2 infection in the study groups. In general, the ARDs group of patients experienced more COVID-19 symptoms than the control group patients. Shortness of breath, fatigue, myalgia, and gastrointestinal symptoms were significantly higher in the ARDs patients than in the control patients (24.8% vs. 10%, p <  0.001; 17.7% vs. 5.8%, p <  0.001; 31.9% vs. 16.8%, p = 0.006; and 20.6% vs. 5.3%, p <  0.001, respectively). Rhinorrhea was the only symptom reported more frequently in the control group than in the ARDs group (2.8% vs. 9.1%, p <  0.001). Severe SARS-CoV-2 infection was more common in the ARDs group at 15% (n = 21) than in the control group at 6% (n = 23) (p <  0.001). The proportions of patients who required oxygen therapy and those who were treated with glucocorticoids as part of a SARS-CoV-2 infection regimen were significantly higher in the ARDs group than in the control group: 19 (13.5%) and 12 (8.5%) vs. 23 (5.8%) and 9 (2.3%) with p = 0.003 and 0.004, respectively. The use of invasive and noninvasive mechanical ventilation was numerically more frequent in the ARDs group but was not significant (7% [5%] vs. 2.8% [11%], p = 0.27). Four deaths were reported within the entire cohort (one in the ARDs group and three in the control group).

### Factors associated with severe SARS-CoV-2 infection in the ARDs group

The 50–65-year age group, use of rituximab, triple therapy, diabetes mellitus, hypertension, cardiovascular disease, chronic kidney disease, and chronic lung disease were significantly associated with severe SARS-CoV-2 infection (p = 0.011, 0.024, 0.042, 0.024, 0.009, 0.002, 0.024, and 0.002, respectively). Table 4 lists the factors associated with severe SARS-CoV-2 infection with corresponding p-values, unadjusted odds ratios, and 95% confidence intervals.

Variables significantly associated with severe SARS-CoV-2 infection in the univariate analysis were included in the multivariate logistic regression analysis. None of the other variables showed a significant and independent association with severe SARS-CoV-2 infection except chronic lung disease (Table 5).

## Discussion

This matched cohort study showed that patients with an ARDs were at a higher risk of severe SARS-CoV-2 infection than those without an ARDs. More patients in the ARDs group required oxygen therapy and glucocorticoids. These findings are in line with those of a report on a large cohort from the United Kingdom, in which patients diagnosed with RA, SLE, or psoriasis and analyzed as a combined group were more likely to die from SARS-CoV-2 than those without these diagnoses^13^. The need for mechanical ventilation was more frequent in the ARDs group, which was also previously shown in a small, matched cohort study from the USA wherein patients with an ARDs required significantly more invasive mechanical ventilation with no difference in mortality rate^14^. However, the need for mechanical ventilation was lower in patients with ARDs in a small study (29 patients with ARDs) from China^15^.

In our study, we identified the risk factors for severe SARS-CoV-2 infection in patients with an ARDs. Some of these factors also applied to the general population, such as diabetes mellitus, hypertension, cardiovascular disease, chronic kidney disease, and chronic lung disease. Other risk factors unique to patients with an ARDs with a potential impact on the severity of SARS-CoV-2 infection were the use of rituximab and triple immunosuppression. Cs-DMARDs and b/ts-DMARDs, which are the most commonly used medications to treat ARDs, were not associated with severe SARS-CoV-2 infection. These findings are supported by those of a large-scale international study using the COVID-19 Global Rheumatology Alliance Registry^16^, which showed that older age, male sex, chronic lung disease, chronic kidney disease, cardiovascular disease combined with hypertension, and moderate/high ARDs activity are associated with higher odds of death. Use of rituximab, sulfasalazine, mycophenolate, cyclosporine, azathioprine, tacrolimus, and glucocorticoids at a prednisolone-equivalent dose >10 mg/day is also associated with higher odds of death compared to methotrexate monotherapy. Interestingly, patients with an ARDs who did not receive a DMAR had higher odds of death than those receiving methotrexate monotherapy^16^. Some of these associations were not detected in our study, possibly due to the smaller sample size. Other reasons that could have led to the low mortality rate in our study were the early detection and treatment of severe COVID-19 cases and the younger age of the study population.

Our study has multiple strengths, including the inclusion of a control group in a matched cohort that accounted for confounders that could affect SARS-CoV-2 outcomes, including age, sex, diabetes mellitus, hypertension, cardiovascular disease, and chronic kidney disease. These factors were well balanced between the two groups. The study population was from a single center with a unified management protocol for SARS-CoV-2 infection, which eliminated inconsistency in the quality of care between the study subjects. Outcome measures were objective and not affected by caregiver decisions or pandemic circumstances, such as hospitalization, which was a commonly used outcome in previous studies. During pandemic peaks, due to the limitations of resources, such as bed capacity, hospitalization criteria could change during pandemic peaks, thereby affecting study outcomes. In contrast, the need for oxygen therapy and ventilation support, as well as the use of glucocorticoids were guided by objective clinical measures. Our definition of severe SARS-CoV-2 infection (oxygen therapy support, invasive or noninvasive ventilation, and use of glucocorticoids) was compatible with the WHO definition, and the use of corticosteroids for severe SARS-CoV-2 infection was recommended in the WHO's September 2020 updated guidelines^17,18^. All SARS-CoV-2 cases were centrally PCR-confirmed at the medical center, which was the sole provider of COVID-19 care in Qatar during the pandemic. In addition, the extensive tracking and testing strategy applied in Qatar allowed us to identify cases with mild or no symptoms. Therefore, our study reflects the true rate of severe SARS-CoV-2 infection. Finally, to the best of our knowledge, this is the first study in our region that has contributed global data on the prognostic factors of SARS-CoV-2 infection in patients with an ARDs.

Our study had limitations that should be considered when interpreting the results, including the lack of laboratory parameters, such as C-reactive protein and ferritin levels. In addition, important factors with a potential impact on the severity of SARS-CoV-2, such as BMI, were missing. Due to the very low mortality rate in Qatar^19^, the sample size was too small to conclude the impact of ARDs on COVID-19-related deaths. Finally, the sample size was inadequate to evaluate the effect of rarer ARDs or less commonly used DMARDs on SARS-CoV-2 infection.

## Conclusions

Patients with an ARDs may have a higher risk of severe SARS-CoV-2 infection than those without an ARDs. Based on univariate and multivariate regression analyses, none of the underlying ARDs or the commonly used ARDs medications, including Cs-DMARDs and cytokine inhibitors, affected the severity of SARS-CoV-2 infection. Furthermore, the use of rituximab or multiple immunosuppressants might be associated with severe infection, which highlights the need for extra caution and close observation of patients with a SARS-CoV-2 infection. Studies with larger sample sizes are needed to further evaluate the factors associated with severe SARS-CoV-2 infection in patients with an ARDs.

## Figures and Tables

**Table 1:** Baseline demographic and clinical characteristics of the study groups

	Cases (n = 141)	Controls (n = 398)	p-value

Sex n (%)			

Female	83 (58.9%)	224 (56.3%)	0.59

Male	58 (41.1%)	174 (43.7%)	

Age, mean (SD)	44.37 (11.41)	43.38 (12.16)	0.4

< 30	13 (9.2%)	53 (13.3%)	

30–49	83 (58.9%)	229 (57.5%)	

50–64	41 (29.1%)	92 (23.1%)	

>65	4 (2.8%)	24 (6.0%)	

Comorbidities			

Hypertension	30 (21.3%)	78 (19.6%)	0.67

Diabetes mellitus	30 (21.3%)	74 (18.6%)	0.36

Cardiovascular disease	5 (3.6%)	6 (1.5%)	0.17

Chronic lung disease	14 (9.9%)	26(6.5%)	0.18

Chronic kidney disease	5 (3.6%)	8 (2.0%)	0.33

Cancer	3 (2.1%)	7 (1.8%)	0.73

Smoker	17 (12.1%)	16 (4.0%)	0.68

WHO regional classification			0.006

AFRO	1 (0.7%)	9 (2.3%)	

EMRO	69 (48.9%)	170 (42.7%)	

EURO	0 (0.0%)	4 (1.0%)	

AMR	0 (0.0%)	3 (0.8%)	

SEARO	68 (48.2%)	180 (45.2%)	

WPRO	0 (0.0%)	32 (8.0%)	

Missing	3 (2.1%)	0 (0.0%)	


WHO, World Health Organization; AFRO, African Region; EMRO, Eastern Mediterranean Regional Office; EURO, European Regional Office; AMR, Region of the Americas; SEARO, South-East Asian Regional Office; WPRO, Western Pacific Regional Office; SD, standard deviation.

**Table 2:** Frequencies of ARDs and the medications used in the study cohort

ARDs	Frequency	ARDs	Frequency	ARDs	Frequency

Connective tissue diseases	43 (30.3%)	Inflammatory arthritis	92 (65.2%)	Others	8 (5.7%)

Systemic lupus erythematosus	16 (11.3%)	Rheumatoid arthritis	57 (40.4%)	Bechet’s disease	3 (2.1%)

Sjogren’s disease	8 (5.6%)	Axial SPA	11 (7.7%)	Uveitis	2 (1.4%)

Antiphospholipid syndrome	15 (10.6%)	Psoriatic arthritis	13 (9.2%)	Gout	2 (1.4%)

Inflammatory myositis	2 (1.2%)	Reactive arthritis	2 (1.4%)	Sarcoidosis	1 (0.7%)

Mixed CTD	2 (1.2%)	Palindromic rheumatism	9 (6.4%)		

Systemic sclerosis	1 (0.7%)				

Undifferentiated CTD	1 (0.7%)				

ANCA associated vasculitis	1 (0.7%)				

Large vessels vasculitis	2 (1.2%)				

b/ts-DMARDs	28 (15.6%)	Cs-DMARDs	95 (67.4%)	Immunosuppression	13 (9.2%)

TNF alfa inhibitor	20 (14.2%)	Hydroxychloroquine	48 (34.0%)	Azathioprine	10 (7.1%)

Rituximab	5 (3.6%)	Methotrexate	45 (31.9%)	Mycophenolate	3 (2.1%)

JAK-i	3 (2.1)	Sulfasalazine	9 (6.4%)	Tacrolimus	2 (1.4%)

		Leflunomide	7 (5%)	Glucocorticoids	18 (12.9%)


ANCA, antineutrophil cytoplasmic antibodies; ARDs, autoimmune rheumatic diseases; b/ts-DMARDs, biological/targeted synthetic disease-modifying antirheumatic diseases; Cs-DMARDs, conventional synthetic disease-modifying anti rheumatic diseases; CTD, connective tissue disease; JAK-i; Janus kinase inhibitor; SPA, spondyloarthropathy; TNF, tumor necrosis factor

**Table 3:** Clinical features of SARS-CoV-2 infection in the study groups

	Cases (n = 141)	Controls (n = 398)	p-value

Symptomatic, n (%)	114 (80.6%)	233 (58.5%)	0.004

Fever	84 (59.6%)	172 (43.2%)	0.088

Sore throat	45 (31.9%)	90 (22.6%)	0.27

Cough	65 (46.1%)	179 (45.0%)	0.19

Shortness of breathing	35 (24.8%)	40 (10.0%)	< 0.001

Fatigue	25 (17.7%)	23 (5.8%)	< 0.001

Myalgia	45 (31.9%)	67 (16.8%)	0.006

Rhinorrhea	4 (2.8%)	39 (9.1%)	< 0.001

Gastrointestinal symptoms	29 (20.6%)	21 (5.3%)	< 0.001

Need for oxygen support	19 (13.5%)	23 (5.8%)	0.003

Need for NIV/InMV support	7 (5.0%)	11 (2.8%)	0.27

Use of glucocorticoids*	12 (8.5%)	9 (2.3%)	0.004

Severe COVID-19 infection**	21 (14.9%)	23 (5.8%)	< 0.001

Death	1 (0.7%)	3 (0.8%)	1


COVID-19, coronavirus disease 2019; InMV, invasive mechanical ventilator; NIV, noninvasive mechanical ventilator. *Use of glucocorticoids as a treatment for SARS-CoV-2 infection. **Severe COVID-19 defined when a SARS-CoV-2-infected patient was managed by oxygen therapy support, invasive or noninvasive mechanical ventilation, or use of glucocorticoids.

**Table 4:** Factors associated with severe and nonsevere SARS-CoV-2 infection

Factors	^*^Severe COVID-19 (n = 21)	Nonsevere COVID-19 (n = 120)	p-value	Unadjusted OR (95% CI)

Sex, n (%)				

Female	10 (47.6%)	73 (60.8%)	0.256	1.709 (0.673–4.337)

Age groups, n (%)				

>65	1 (4.8%)	3 (2.5%)	0.479	1.950 (0.193–19.691)

50–65	11 (52.7%)	30 (25.0%)	0.011	0.303 (0.117–0.784)

30–49	9 (42.9%)	74 (61.7%)	0.106	2.145 (0.838–5.487)

< 30	0 (0)	13 (10.8%)	0.216	0.836 (0.774–0.903)

Inflammatory arthritis, n (%)	14 (66.7%)	78 (65.0%)	0.882	1.077 (0.403–2.875)

Rheumatoid arthritis	12 (57.1%)	45 (37.5%)	0.091	2.222 (0.868–5.689)

Spondyloarthropathy	1 (4.8%)	25 (20.8%)	0.124	0.190 (0.024–1.485)

Connective tissue disease, n (%)	8 (38.1%)	35 (29.2%)	0.412	1.495 (0.570–3.922)

Systemic lupus erythematosus	2 (9.5%)	14 (11.7%)	1	0.797 (0.167–3.793)

Antiphospholipid syndrome	3 (14.3%)	12 (10.0%)	0.47	1.500 (0.385–5.844)

Sjogren’s disease	1 (4.8%)	7 (5.8%)	1	0.807 (0.094–6.919)

Not on ARDs medications, n (%)	4 (19.0%)	20 (16.7%)	0.758	1.176 (0.358–3.868)

Cs-DMARDs, n (%)	14 (9.9%)	81 (57.1%)	0.94	0.963 (0.360–2.577)

Hydroxychloroquine	6 (28.6%)	42 (35.0%)	0.566	0.743 (0.268–2.057)

Methotrexate	9 (42.9%)	36 (30.0%)	0.244	1.750 (0.678–4.518)

Leflunomide	1 (4.8%)	6 (5.0%)	1	0.950 (0.109–8.318)

Sulfasalazine	2 (9.5%)	7 (5.8%)	0.623	1.699 (0.328–8.803)

b/ts-DMARDs, n (%)	6 (28.6%)	22 (18.3%)	0.278	1.782 (0.621–5.110)

TNF alfa inhibitors	2 (9.5%)	18 (15.0%)	0.738	0.596 (0.128–2.785)

Rituximab	3 (14.3%)	2 (1.7%)	0.024	9.833 (1.536–62.956)

JAK-i	1 (4.8%)	2 (1.7%)	0.386	2.950 (0.255–34.076)

Immunosuppression, n (%)	2 (9.5%)	11(9.2%)	1	1.043 (0.214–5.082)

Azathioprine	1 (4.8%)	9 (7.5%)	1	0.617 (0.074–5.138)

Glucocorticoids	4 (19.0%)	14 (11.7%)	0.475	1.782 (0.524–6.056)

Monotherapy, n (%)	9 (42.9%)	68 (56.7%)	0.241	0.574 (0.225–1.463)

Dual therapy, n (%)	4 (19.0%)	26 (21.7%)	1	0.851 (0.263–2.748)

**Triple therapy, n (%)	4 (19.0%)	6 (5.0%)	0.042	4.471 (1.143–17.487)

Remission/Low disease activity	17 (81.0%)	103 (85.8%)	0.562	0.701 (0.210–2.338)

Moderate/high disease activity	4 (19.0%)	14 (11.7%)	0.475	1.782 (0.524–6.056)

Comorbidities, n (%)				

Diabetes mellitus	9 (42.9%)	21 (17.5%)	0.009	3.536 (1.321–9.461)

Hypertension	9 (42.9%)	21 (17.5%)	0.009	3.536 (1.321–9.461)

Cardiovascular disease	4 (19.0%)	1 (0.8%)	0.002	28.0 (2.953–265.513)

Chronic kidney disease	3 (14.3%)	2 (1.7%)	0.024	9.833 (1.536–62.956)

Chronic lung disease	6 (28.6%)	8 (6.7%)	0.002	5.600 (1.707–18.)


ARDs, autoimmune rheumatic diseases; b/ts-DMARDs, biological/targeted synthetic disease-modifying anti rheumatic diseases; CI, confidence interval; COVID-19, coronavirus disease 2019; Cs-DMARDs, conventional synthetic disease-modifying anti rheumatic diseases; JAK-i; Janus kinase inhibitor; OR, odds ratio; TNF, tumor necrosis factor.* severe infection was defined when a SARS-CoV-2-infected patient was managed with oxygen therapy support, invasive or noninvasive ventilation, or glucocorticoids.** ≥ 3 drugs.

**Table 5:** Multivariate logistic regression analysis for severe SARS-CoV-2 infection

Predictors	p-value	Adjusted Odds ratio	95% confidence interval

Age group 50–64 years	0.194	2.159	0.677–6.891

Diabetes mellitus	0.153	2.423	0.720–8.151

Hypertension	0.696	1.316	0.332–5.220

Cardiovascular disease	0.074	10.563	0.793–140.762

Chronic lung disease	0.024	4.99	1.238–20.108

Chronic kidney disease	0.369	3.034	0.269–34.191

Rituximab	0.359	2.755	0.315–24.063

Triple therapy	0.274	2.785	0.443–17.478


Chronic lung disease was independently associated with severe SARS-CoV-2 infection. None of the other variables was independently associated with severe SARS-CoV-2 infection

## References

[bib1] Cobb S, Anderson F, Bauer W. (1953). Length of life and cause of death in rheumatoid arthritis. N Engl J Med.

[bib2] Listing J, Gerhold K, Zink A. (2013). The risk of infections associated with rheumatoid arthritis, with its comorbidity and treatment. Rheumatology (Oxford).

[bib3] Souza DC, Santo AH, Sato EI. (2012). Mortality profile related to systemic lupus erythematosus: a multiple cause-of-death analysis. J Rheumatol.

[bib4] Al Kuwari HM, Abdul Rahim HF, Abu-Raddad LJ, Abou-Samra AB, Al Kanaani Z, Al Khal A, et al. (2020). Epidemiological investigation of the first 5685 cases of SARS-CoV-2 infection in Qatar, 28 February–18 April 2020. BMJ Open.

[bib5] Hanif M, Haider MA, Xi Q, Ali MJ, Ahmed MU. (2020). A review of the risk factors associated with poor outcomes in patients with coronavirus disease 2019. Cureus.

[bib6] Hussain A, Mahawar K, Xia Z, Yang W, El-Hasani S. (2020). Obesity and mortality of COVID-19.Meta-analysis. Obes Res Clin Pract.

[bib7] Lokken EM, Huebner EM, Taylor GG, Hendrickson S, Vanderhoeven J, Kachikis A, et al. (2021). Disease severity, pregnancy outcomes, and maternal deaths among pregnant patients with severe acute respiratory syndrome coronavirus 2 infection in Washington State. Am J Obstet Gynecol.

[bib8] Omrani AS, Almaslamani MA, Daghfal J, Alattar RA, Elgara M, Shaar SH, et al. (2020). The first consecutive 5000 patients with Coronavirus Disease 2019 from Qatar; a nation-wide cohort study. BMC Infect Dis.

[bib9] Pranata R, Supriyadi R, Huang I, Permana H, Lim MA, Yonas E, et al. The association between chronic kidney disease and new onset renal replacement therapy on the outcome of COVID-19 patients: a meta-analysis. Clin Med Insights Circ Respir Pulm Med.

[bib10] World Health Organization. WHO Coronavirus (COVID-19) Dashboard. https://covid19.who.int/?adgroupsurvey.

[bib11] Gianfrancesco MA, Hyrich KL, Gossec L, Strangfeld A, Carmona L, Mateus EF, et al. (2020). Rheumatic disease and COVID-19: initial data from the COVID-19 Global Rheumatology Alliance provider registries. Lancet Rheumatol.

[bib12] Liew JW, Bhana S, Costello W, Hausmann JS, Machado PM, Robinson PC, et al. (2021). The COVID-19 Global Rheumatology Alliance: evaluating the rapid design and implementation of an international registry against best practice. Rheumatology (Oxford).

[bib13] Williamson EJ, Walker AJ, Bhaskaran K, Bacon S, Bates C, Morton CE, et al. (2020). Factors associated with COVID-19-related death using OpenSAFELY. Nature.

[bib14] D'Silva KM, Serling-Boyd N, Wallwork R, Hsu T, Fu X, Gravallese EM, et al. (2020;). Clinical characteristics and outcomes of patients with coronavirus disease 2019 (COVID-19) and rheumatic disease: a comparative cohort study from a US ‘hot spot’. Ann Rheum Dis.

[bib15] Zhao J, Pang R, Wu J, Guo Y, Yang Y, Zhang L, et al. (2021). Clinical characteristics and outcomes of patients with COVID-19 and rheumatic disease in China ‘hot spot’ versus in US ‘hot spot’: similarities and differences. Ann Rheum Dis.

[bib16] Strangfeld A, Schäfer M, Gianfrancesco MA, Lawson-Tovey S, Liew JW, Ljung L, et al. (2021). Factors associated with COVID-19-related death in people with rheumatic diseases: results from the COVID-19 Global Rheumatology Alliance physician-reported registry. Ann Rheum Dis.

[bib17] World Health Organization. (2020.). Corticosteroids for COVID-19. https://www.who.int/publications/i/item/who-2019-ncov-corticosteroids-2020.1.accessed.

[bib18] World Health Organization. Clinical management of COVID-19 - interim guidance 27 May 2020. https://apps.who.int/iris/rest/bitstreams/1278777/retrieve.

[bib19] Alwahaibi N, Al Maskari M, Al Dhahli B, Al Issaei H, Al-Jaaidi Shadia Al Bahlani S. One-year review of COVID-19 in the Arab world. Qatar Med J.

